# Data providing detailed county level information for eWIC rollout in Ohio

**DOI:** 10.1016/j.dib.2019.103955

**Published:** 2019-04-27

**Authors:** Andrew S. Hanks, Carolyn Gunther, Dean Lillard, Robert Scharff

**Affiliations:** a Department of Human Sciences, College of Education and Human Ecology, The Ohio State University, USA; b Deutsches Institut für Wirtschaftsforschung – Berlin (DIW-Berlin), National Bureau of Economic Research, Cambridge, MA, USA

## Abstract

By October 2020, states across the nation must deliver benefits for the WIC program via electronic benefits transfer, also referred to as eWIC. The state of Ohio made the transition from 2014 to 2015 and staggered implementation across counties. In this article, we present county-level data on the specific dates Ohio counties changed to eWIC. We also present detailed demographic data for the counties included in each roll-out date.

**Table:** Specifications table

Subject area	*Economics*
More specific subject area	*Economics of Nutrition Assistance Programs*
Type of data	*Tables*
How data was acquired	*State and federal government websites*
Data format	*Analyzed*
Experimental factors	*Counties in Ohio introduced eWIC to facilitate benefit redemption for WIC recipients.*
Experimental features	*The Ohio Department of Health staggered eWIC implementation in the counties across seven different phases from July 2014 through July 2015.*
Data source location	*We collected eWIC implementation dates from newsletters published by the Ohio Department of Health, which we accessed online. We collected county demographic information from publicly available files online.*
Data accessibility	*We include all data with this article.*
Related research article	*Hanks, Andrew S., Carolyn Gunther, Dean Lillard, and Robert Scharff. (2018) From Paper to Plastic: Understanding the Impact of eWIC on WIC Recipient Behavior. Food Policy. In Press.**[1]*

**Table:** 

**Value of the data** • Data on eWIC implementation dates in Ohio counties listed in Table 1 shows the exact sources of variation in eWIC implementation. Researchers can use this variation in Ohio, and tap into similar variation in other states, to identify the impact of eWIC on WIC recipient shopping behavior. • The staggered implementation dates also provide a good source of variation for researchers to study the potential effect EBT has on enrollment in WIC • Detailed county-level data in Table 2 show similarities and differences in demographic characteristics across each eWIC rollout phase. This may help researchers identify region specific interventions for underprivileged and/or low-income households.

## 1 Data

State officials in the Ohio Department of Heath (ODH)adopted a staggered implementation approach to roll out eWIC technology across all 88 counties. In 2014, state officials piloted eWIC technology in 5 counties in three different phases. In 2015, the state completed the rollout to the remaining 83 counties in four separate phases (Table 1). Additional details of the rollout can be found in [1]. We also collected detailed demographic data for each county in Ohio and aggregated these data by roll out phase. We report these data in Table 2. The authors will provide copies of the newsletters and their county-level demographic data up on request.

**Table 1:** Timeline for WIC electronic benefits transfer transition in Ohio.

Pilot Studies	January 26, 2015	March 23, 2015	May 1, 2015	July 1, 2015
Jul 14, 2014^a^	Athens	Belmont	Adams/Brown	Ashtabula
Licking	Gallia	Carroll	Allen	Auglaize
Aug 4, 2014^a^	Jackson	Coshocton	Champaign	Butler
Greene	Noble	Fairfield	Clark	Crawford
Oct 19, 2014^a^	Pike	Guernsey	Clermont	Cuyahoga
Hocking	Vinton	Harrison	Clinton	Darke/Mercer
Meigs, Putnam	Washington/Morgan	Holmes	Defiance	Erie/Huron
		Jefferson	Delaware/Morrow/Union	Fulton/Henry
		Lawrence	Fayette	Geauga
		Monroe	Franklin	Hamilton
		Muskingum	Hancock/Hardin	Knox
		Perry	Highland	Lake
		Ross/Pickaway	Logan	Lorain
		Scioto	Lucas	Mahoning
		Tuscarawas	Madison	Marion
			Montgomery	Medina
			Ottawa	Miami
			Paulding	Preble
			Shelby	Portage/Columbiana
			Warren	Richland/Ashland
			Williams	Sandusky
			Wood	Seneca
			Wyandot	Stark
				Summit
				Trumbull
				Van Wert
				Wayne

a These are the three pilot phase dates. In the October pilot, the EBT transition occurred sometime during the week of the 19th.

**Table 2:** County Demographics by eWIC Rollout Phase and by Inclusion in the sample of Transaction Data.

	Pilots	January 26, 2015	March 23, 2015	May 1, 2015	July 1, 2015
*Population*	83,891	32,599	59,593	143,723	189,429
Percent white	91%	95%	94%	80%	81%
Percent black	4%	2%	3%	13%	14%
*Households*	19,062	6584	13,717	34,120	42,527
Percent with welfare income	26%	38%	31%	29%	30%
*Families*	18,949	6510	13,590	33,827	42,153
Percent with welfare income	25%	37%	31%	29%	30%
Percent married couples	72%	66%	70%	64%	63%
With welfare income	11%	16%	13%	10%	9%
Percent single male head	6%	12%	8%	8%	7%
With welfare income	2%	7%	3%	3%	3%
Percent single female head	21%	22%	23%	28%	29%
With welfare income	12%	14%	14%	16%	18%
*Median household income ($2015)*	$51,434	$39,237	$45,048	$51,131	$49,716
Households with SNAP	$17,477	$15,763	$17,046	$18,479	$18,428
Households without SNAP	$58,318	$46,943	$52,038	$56,834	$55,372
Number of counties	5	8	16	27	32

## 2 Experimental design, materials and methods

To retrieve the eWIC implementation data for Ohio we extracted information from newsletters supplied available on the Ohio Department of Health's website. ODH publishes these newsletters for WIC recipients to keep them informed about WIC program updates and changes. These newsletters contain detailed information about eWIC introduction in counties, how recipients can obtain eWIC cards, and how they redeem benefits with eWIC.

To identify potential setbacks during implementation, ODH officials used three different pilot phases in 2014 (see Table 1) with counties of various sizes and demographic characteristics. Once ODH worked through issues identified in the pilot phases, it implemented eWIC in eight relatively smaller counties on January 26, 2015 and gradually increased the number of counties (see Table 1), and average county population (see Table 2), in implementation groups in the remaining three phases.

Table 2 also provides data on income, household size, and poverty for the groups of counties in each implementation date. We retrieved these data from the Census website [2] and generated data in Table 2 by averaging data across all counties in each roll-out phase. Counties that transitioned in January 26 had the lowest household median income overall, and for households that participated in the Supplemental Nutrition Assistance Program (SNAP). This group of eight counties also had, on average, the fewest households and the largest percentage of households with welfare income. Figure 1 in [1] graphically illustrates how ODH geographically grouped counties for implementation. Counties that implemented eWIC on January 26 are all in the Appalachian region.
