# Supplementary material for: Data providing detailed county level information for eWIC rollout in Ohio
Source: Data Brief. 2019 Apr 27;24:103955. doi: 10.1016/j.dib.2019.103955 (PMC6517565; doi:10.1016/j.dib.2019.103955)
Supplement: Multimedia component 1 [file mmc1.docx]

Conflicts of interest

All authors declare no conflicts of interest related to the research in this manuscript.
